# A Confirmation Bias View on Social Media Induced Polarisation During Covid-19

**DOI:** 10.1007/s10796-021-10222-9

**Published:** 2021-11-20

**Authors:** Sachin Modgil, Rohit Kumar Singh, Shivam Gupta, Denis Dennehy

**Affiliations:** 1 International Management Institute (IMI) Kolkata, Kolkata, India; 2 NEOMA Business School, Mont-Saint-Aignan, France; 3 National University of Ireland Galway, Galway, Ireland

**Keywords:** Echo Chambers, Social Media Induced Polarisation, Confirmation Bias, Covid-19

## Abstract

Social media has played a pivotal role in polarising views on politics, climate change, and more recently, the Covid-19 pandemic. Social media induced polarisation (SMIP) poses serious challenges to society as it could enable ‘digital wildfires’ that can wreak havoc worldwide. While the effects of SMIP have been extensively studied, there is limited understanding of the interplay between two key components of this phenomenon: confirmation bias (reinforcing one’s attitudes and beliefs) and echo chambers (i.e., hear their own voice). This paper addresses this knowledge deficit by exploring how manifestations of confirmation bias contributed to the development of ‘echo chambers’ at the height of the Covid-19 pandemic. Thematic analysis of data collected from 35 participants involved in supply chain information processing forms the basis of a conceptual model of SMIP and four key cross-cutting propositions emerging from the data that have implications for research and practice.

## Introduction

Social media is increasingly being used by organisations to manage business activities and relationships among supply-chain stakeholders (Choi et al., 2020; Huang et al., 2020; Singh et al., 2018) including, new product development (Giannakis et al., 2020), service improvement (Fan & Niu, 2016), daily sales forecasts (Cui et al., 2019), and information dissemination (Kanagarajoo et al., 2019). The prevalence and speed at which social media platforms (e.g., WeChat, Facebook, LinkedIn, Twitter, Flickr, WhatsApp) facilitate the exchange of real-time data (Gorwa & Guilbeault, 2020; Rousidis et al., 2020) has made it a popular tool for effective supply chain management (Choi et al., 2020). For instance, throughout the supply chain, social media is being used to monitor in real-time, risks, transactions, consumer sentiment, and purchasing decisions, as well as interact with consumers to track their behaviour (Brusset & Teller, 2017; Havakhor et al., 2018; Min & Zhou, 2002). Essentially, social media enables stakeholders of a supply chain to communicate and exchange information that can impact the forecasting of disruptions, order fulfilment, and production flow to match supply and demand (Grant, 2016; Rauniar et al., 2014). On one hand social media helps in communicating individuals and organizations about their products, services and developing network of communities that can be helpful to share the information on real time basis and create the awareness on a specific issue such as health during Covid-19 (Mheidly & Fares, 2020). On the other hand, social media is full of possibilities of fake and misleading content such as Covid-19 variants and their cure along with role of vaccination in fighting Covid-19, along with cyber risks (Brummette et al., 2018; Islam et al., 2020).

The universal access to real-time information that social media provides to users (Dwivedi et al., 2018) can lead to supply chain participants becoming highly susceptible to ‘confirmation bias’ and ‘echo chambers’ e.g., situations where individuals “hear their own voice” (Bessi, 2016; Boutyline & Willer, 2017; Brugnoli et al., 2019; Jacobson et al., 2016). The influence of echo chambers includes excluding alternate perspectives (Gillespie et al., 2014), a lack of common ground (Layman et al., 2006) and political, administrative, and social gridlock in many contexts (Hutchens et al., 2021; Kim & Kim, 2019). Therefore, an approach of mindfulness and reliability needs to be developed to avoid echo chambers across the supply chain (Butler & Gray, 2006; Dernbecher & Beck, 2017; Thatcher et al., 2018). Hence, social media induced polarization (SMIP) can be defined as skewed opinions, information/misinformation regarding socio-cultural activities including products or services that can lead to mass destruction of property and other crisis (O’Hara & Stevens, 2015).

The devastating effect of SMIP on communities, businesses, and governments has been identified by the World Economic Forum (2018) as the greatest threat to society due to the speed at which ‘digital wildfires’ spread on a global scale. Discourse on SMIP has reached mainstream media with prominent business leaders including Tim Cook (CEO, Apple) criticising polarisation and misinformation due to social media and the facilitating role that technology companies play by prioritising conspiracy theories and violent incitement because of their high rates of engagement (Reuters, 2021; Weckler, 2021). The providers as well as users are the victims of unreliable information that affect the decision making for most of the organizations.

In the context of SMIP, echo chambers refer to situations where users consume content and engage in discourse that predominantly supports the same point of view that users hold and believe themselves (Boutyline & Willer, 2017; Flaxman et al., 2016). In the context of this study, social media can induce polarisation in the form of echo chambers in global supply chains due to regional differences in terms of selecting, filtering, and sorting of information on social media (Flaxman et al., 2016; Miroudot, 2020). For example, echo chambers can occur as a global event unfolds (i.e., Covid-19, natural disaster) whereby supply chain participants are influenced by their professional and personal experiences (Bhagwat & Sharma, 2007; Muckstadt et al., 2001), as well as the information that is circulated and referred through social media (Gupta et al., 2021; Barberá et al., 2015; Toubiana & Zietsma, 2017). The impact of echo chambers on supply chain decision making can lead to excessive or insufficient stock levels in regional warehouses (Sharma et al., 2020).

The ubiquitous nature and speed of social media has been attributed to its popularity as a tool to coordinate supply chain activities (Choi et al., 2020). The speed of information dissemination on social media is also highly influential as content published first tends to get more attention and it influences the opinion of end users (Roozenbeek & van der Linden, 2019; Wang et al., 2015), as well as decision makers spread across the globe (Arnott, 2006; Wu et al., 2019). Supply chain participants are also increasingly exposed to high volumes of information and over a period they tend to favour certain social media platforms due to their familiarity with the source of information rather than its trustworthiness (Fidel & Green, 2004; Orji et al., 2020). The choice of social media platform can significantly impact supply chains from a risk management perspective (Culp, 2020; Fu et al., 2013; O’Leary, 2011) as supply chain participants are influenced by the sentiment of posts, concerns or tweets that are expressed by customers, retailers, and other stakeholders (Clapham et al., 2019; Wuebben, 2016; Zadeh & Sharda, 2014).

As supply chains become increasingly digitised using social media platforms and other digital technologies (i.e., AI, Big data), supply chain participants are increasingly seeking and believing the information from like-minded individuals (He et al., 2017; Sestino et al., 2020; Tim et al., 2021). This approach can increase the risk to echo chambers as participants may unknowingly seek reinforcement of existing biases and beliefs (Bhardwaj et al., 2016; Flaxman et al., 2016).

The phenomenon of ‘SMIP’ has been studied from different perspectives (e.g., Flaxman et al., 2016; Geschke et al., 2019; O’Hara & Stevens, 2015; Spohr, 2017) and in varied contexts, such as politics (Kim & Kim, 2019; Hyun, & Moon, 2016; Yarchi et al., 2020, Lee, 2016), climate change (e.g., Fisher et al., 2013; Leviston et al., 2013), and news framing (Han & Federico, 2018; Hyun & Moon, 2016). Recent studies (e.g., Del Vicario et al., 2017; Sikder et al., 2020) have begun modelling the combination of bias and polarisation to examine the impact of misinformation in social media networks. There remains however, much scope to advance understanding of SMIP as there is a noticeable absence of research that focuses on the structure of social media polarisation in the context of global events such as Covid-19, where participants are highly susceptible to developing echo chambers through confirmation bias (Boutyline & Willer, 2017; Orji et al., 2020). To this end, the aim of this study is to *‘explore how manifestations of confirmation bias contribute to the development of echo chambers, in the context of supply chains during the Covid-19 pandemic*.’ To achieve this aim, we seek to answer the following research question: *How do the elements of SMIP lead to the emergence of echo chambers in the context of supply chains?*

To answer this research question, this study adopts an interpretive qualitative approach to explore SMIP from the perspective of supply chain participants by using thematic analysis to identify how four elements (e.g., environmental setting, actors, mechanism, and outcome) contribute to the development of echo chambers.

The remainder of the paper is structured as follows. A review of pertinent SMIP literature that informs our conceptualisation of echo chambers through confirmation bias is presented. Next, the research method and data collection and analysis are outlined. Then key findings and emergent themes are presented that form the basis of four cross-cutting propositions. Followed by a discussion, implications, and future research. The paper ends with a conclusion.

## Literature Review

### Social Media Induced Polarisation

The proliferation of social media has been a catalyst to inducing a dangerous, socio-cultural polarisation of society (Montalvo & Reynal-Querol, 2005; Spohr, 2017; Vishwanath, 2015). This polarisation phenomenon has led researchers to explore SMIP from different theoretical lenses to study specific social media platforms and particular attention given to fake news (Langley et al., 2021; Brummette et al., 2018; Lee et al., 2015a; Shearer & Grieco, 2019; Singh et al., 2020). Manifestations of SMIP include misinformation about presidential election campaigns (Guess et al., 2018; Linvill & Warren, 2020; Schäfer et al., 2017), race (Jamieson, 2020), immigration (Jaramillo-Dent & Pérez-Rodríguez, 2021; Newman et al., 2017), religion (Said, 2008), and pandemics, specifically Covid-19 (Laato et al., 2020). The rapid global adoption of social media technology has significantly influenced many job roles and supply chain activities (Ribarsky et al., 2014; Zamani et al., 2020). Organisations are incorporating social media technology as a core capability to deliver information efficiently and cost-effectively to stakeholders in their supply chain (Osatuyi, 2013; Thatcher et al., 2018; Xie & Lee, 2015; Zhang et al., 2017). For example, increasing its visibility in the supply chain ecosystem, facilitating real-time communication between its customers, and to mitigate risks (Wu & Li, 2018).

Social media tools (e.g., blogs, wikis) and platforms (Facebook, Twitter, and LinkedIn etc.) are increasingly being used by consumers (individuals) and providers (organisations) to (i) post and share information, (ii) develop a network of communities, and to achieve competitive advantage in both developed and developing countries (Batrinca & Treleaven, 2015; Obar et al., 2012; Stieglitz & Dang-Xuan, 2013). Essentially, social media enables individuals and organisations to act as influencers in the dissemination of information in terms of posting, re-sharing, commenting, tagging, and re-tweeting (Lee et al., 2015b; Shin & Thorson, 2017). The dark side of this form of endorsed information sharing is that consumers of the information are exposed to ‘filtered’ information (Fletcher & Nielsen, 2018; Gallaugher & Ransbotham, 2010; Kietzmann et al., 2011; Tran et al., 2020). This exposure increases the risk of misinformation being shared across the social media platform, leading to SMIP, such as echo chambers (Brugnoli et al., 2019; Mondal et al., 2018; Toubiana & Zietsma, 2017; Wu et al., 2019). Therefore, individuals need to rely on mindfulness and adopt reliable technologies consuming and sharing information (Endsley, 2018). Individuals also tend to seek social media platforms that support their beliefs and attitudes, to gain confidence in their biased views (Arnott, 2006; Nikolov et al., 2019; Shin & Thorson, 2017; Susarla et al., 2012). The fake content circulating on social media lead to extreme situations. For example, in 2019, a baby’s bloodied corpse led to the violence and killed at least 10 people in Africa’s Nigeria (LA Times, 2019). In another fake campaign run by secondary school students in Chile against the fare of metro, led to the developments of civil protest, violence and deaths leading to the instability for weeks in the country (Atlantic Council, 2019). Recently, people are posting the content related to Covid-19 and its vaccination impact and people are avoiding taking the vaccine, which is culminating the fear of another variant of Covid-19 and resulting into slow progress of inoculation (India Today, 2021).

The degree of SMIP is however, depends on the type of network (e.g., centralised or egalitarian) that individual consumers have joined (Juris, 2012). In a centralised network there is usually only one or a select number of individuals at the centre who play the role of ‘influencer’ as they can filter or block information before sharing it to a much wider network of its members (Culnan et al., 2010; Gallaugher & Ransbotham, 2010). While in an egalitarian network, all members have equal contact and influence across the network and therefore opinions can be shared by any individual within the network, and information is shared unfiltered to all members (Sikder et al., 2020). The attitude or belief system of a participant can be influenced due to the information processing approaches in both types of networks (Petty et al., 2009). When a participant is motivated and capable, they will carefully engage, reflect, and connect with pre-existing beliefs and then influence the cognitive network (Arnott, 2006). A cognitive network indicates a complex structure, where nodes signify concepts and branches specify one or more type of conceptual relationship, for instance two words describe the same sense in one context or like one another (Islam et al., 2020). The cognitive network is dependent on cognitive ability of individuals those are motivated, not motivated or lack cognitive ability may engage in peripheral processing and rely on the source of information due to its credibility that can lead to a change of attitude (Petty et al., 1987). Compared to central processing, the egalitarian network does not produce long lasting changes in belief system (Kelman, 2006).

SMIP can be witnessed in both types of networks (Centralised and Egalitarian) as partisan bias can become amplified when bias is displayed by a centralised echo chamber, while in the egalitarian echo chambers, the quality of ideas is a basis for their spread as compared to a particular individual (Chou et al., 2018). In the context of supply chains, participants act as ‘partisan’ (e.g., creator and gatekeeper of the shared information) or ‘bipartisan’ (e.g., consumer of the information or reshare/endorse the information) on social media platforms (Anand et al., 2007; Sharif, 2002). For example, due to national and regional lockdowns during the Covid-19 pandemic, people spent more time on social media discussing their views about the virus and vaccines (Sarkis, 2020; Islam et al., 2020; Nabity-Grover, 2020). Using social media in this way is an ‘infodemic’, due to the creation and consumption of misinformation related to the virus and vaccines which created confusion and distrust among people and hampered an effective response to the pandemic (Verma & Gustafsson, 2020) and influencing the supply chains.

### Confirmation Bias

Confirmation bias occurs, when users encounter information that reinforces their pre-existing beliefs and attitudes (Bessi, 2016; Geschke et al., 2019; Zhao et al., 2020). Confirmation bias is defined as the tendency to interpret, search for, recall, and favour the information in a way that confirms one’s pre-existing beliefs or hypotheses resulting into polarised views about an issue or event (Westerwick et al., 2017; Gupta et al., 2021). Confirmation biases indicate why a group of individuals with opposing views on a topic can view the same evidence. The individuals those are victims of confirmation bias give more weightage to evidence to support their beliefs rather than undervaluing the evidence that can disprove it (Westerwick et al., 2017; Huang et al., 2012). Therefore, the tendency of people to favour the information on their prior exposure or hypotheses can be regarded as confirmation bias, which is stronger for the activities and issues those are highly emotive and deeply entrenched (Zhao et al., 2020). The information presented on social media is not only reflective of the designer’s values and beliefs, which can be one-sided and/or sensational (Zhang et al., 2017; Pariser, 2011). Previous studies conclude that users generally tend to search, believe, and share information that conforms to their own beliefs and attitudes, but ignore the information that challenges their beliefs and attitudes (Itzchakov & Van Harreveld, 2018; Knobloch-Westerwick & Kleinman, 2012; van Strien, Kammerer et al., 2016). In the context of SMIP, social media platforms facilitate the configuration and aggregation of ‘like-minded’ people and network that reinforce and further empower polarised groups (Giannoccaro, 2018; Westerwick et al., 2017; Stroud, 2008).

### Echo Chambers

Echo chambers refer to the situation where people ‘hear their own voice’ (Boutyline & Willer, 2017; Flaxman et al., 2016). In the context of social media, it refers to situations where users consume content that expresses the same point of view that users hold themselves (Bessi, 2016; Geschke et al., 2019). As social media platforms largely rely on algorithms to generate large quantity of content, which can lead to the emergence of conspiracy theories, other forms of distorted information, and even extremist groups with a shared ideology, which further lead to the emergence of echo chambers (Boutyline & Willer, 2017; Flaxman et al., 2016; Schilling & Fang, 2014). The evolution of a group or community can be observed through the configuration of communication patterns (Tim et al., 2018). Weak patterns of communication between a group of users can be observed as compared to strong patterns of communication within the group, leading to the formation of echo chambers (Brugnoli et al., 2019). Since it is difficult to further identify the sub-groups by just analyzing the communication pattern on social media platforms, the measurement of echo chambers cannot be identified at group level as it requires the measurement of communication pattern within group as compared to between groups (Carr & Hayes, 2015; Go & You, 2016). However, the level of fragmentation between groups can define the degree of echo in chambers (Brugnoli et al., 2019).

**Fig. 1:**
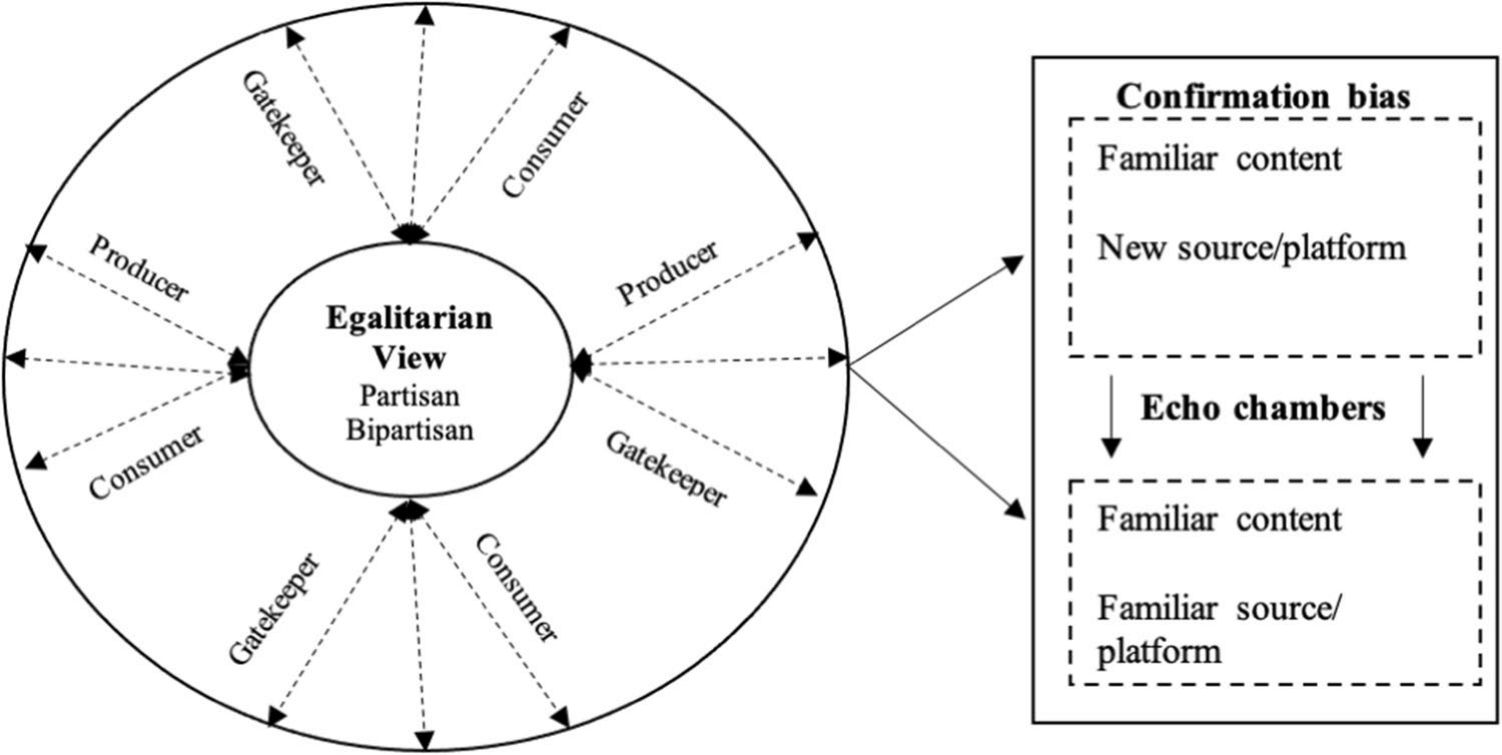
Conceptualisation of echo chambers through confirmation bias on social media

The exponential growth of data creation, data flow and consumption across the spectrum of social media platforms offers many opportunities and challenges for supply chain participants (Legner et al., 2017; Sarimveis et al., 2008). On the one hand, supply chain participants are actively looking for the modes, where they can capture the precise information and improve the visibility, control, and efficiency in their supply chain (Sodhi & Tang, 2019). On the other hand, supply chain activities are governed by participants who have their own pre-existing beliefs and attitudes that can lead to ‘confirmation bias’, which can negatively impact the supply chain activities (Huang et al., 2020; Huang et al., 2012; Zhao et al., 2020). Earlier studies on social media have majorly focused on identity, social power, social capital, and social loafing that led to polarisation based on individuals’ attitudes, actions, and intentions for using social media (Dwivedi et al., 2018; Erkan & Evans, 2016; O’Leary, 2011; Rauniar et al., 2014; Rim et al., 2020). Indeed, such studies have made valuable contributions to knowledge; however, they do not conceptualise how the (i) foundational elements, (ii) role of actors, and (iii) phenomena lead to the emergence of echo chambers through dominance of confirmation bias (Boulianne et al., 2020; Sasahara et al., 2020). In this study, we conceptualize how confirmation bias can illuminate elements that can contribute to the development of ‘echo chambers’ in the context of supply chains (see Fig. 1).

In the context of supply chains, participants include professionals, consulting firms and organisations that play an equal role in creating and sharing the information. It is therefore appropriate to view the system as egalitarian, where partisan and bi-partisan members co-exist. Partisans are the producers and users of the content with one-sided leaning, whereas bi-partisan producers create and consume the content with both side view. The gatekeeper type of users consumes the content with both perspectives but produce it with single sided leaning after some filtration. These consumers and producers represent a smaller group with higher network centrality, without much embedded in their community network. It is relatively easy to identify partisan users as compared to gatekeepers (Garimella et al., 2018). The characteristics of partisans, bi-partisans and gatekeepers leads to the development of confirmation bias, whereby content is related and familiar and posted through new source (Jacobson et al., 2016). When different users on social media consume the information from different sources, they develop the echo chambers on the premise of confirmation of their beliefs resulting into polarisation.

## Research Methods

**Fig. 2:**
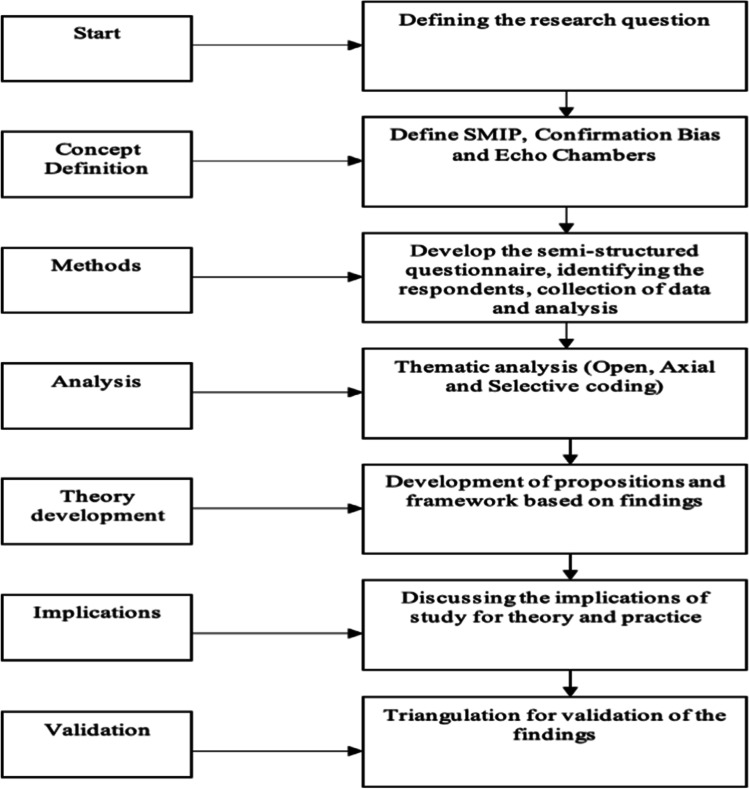
Research design

This exploratory study adopts an interpretive case study approach as it is suited to (i) understanding of the influence of context (Walsham, 1993), (ii) examining a new phenomenon that lacks empirical substantiation (Benbasat et al., 1987; Eisenhardt, 1989; Stake, 2000), and (iii) extending the theoretical underpinning of the study (Benbasat et al., 1987; Pagell & Wu, 2009; Stuart et al., 2002). The research design (see Fig. 2) provides a clear ‘chain of evidence’ (Yin, 2002) with explicit links between the research questions, the data collected, and the conclusions drawn, which helps “follow the derivation of any evidence, ranging from initial research questions to ultimate case study conclusions.” (Yin, 2002, p.83).

### Data Collection

Using snowball sampling (Naderifar et al., 2017; Noy 2008), 35 supply chain practitioners with extensive industry experience and knowledge of AI were identified for the purpose of this study (see Table 1). Interviews were conducted between September and December 2020. Due to Covid-19, interviews were conducted remotely via Microsoft Teams and Zoom. The duration of interviews ranged between 30 and 50 min. Interviews were recorded and transcribed using Otter, an AI-based transcription software, proof-read and annotated. Data saturation (Birks et al., 2013; Nelson, 2017) was achieved after 35 interviews, and therefore, no additional interviews were conducted. For the purpose of research rigour and anoymonity, a code (e.g., R1 to R35) was assigned to each interviewee.

**Table 1:** Interviewee profile

No.	Code	Job title	Nature of work	Years of Experience
1	R1	Senior Manager	Sales & Marketing	>10
2	R2	Senior Manager	Logistics & Supply Chain	>10
3	R3	Marketing Executive	Sales & Marketing	6-10
4	R4	Logistics Manager	Sales & Marketing	6-10
5	R5	Corporate Finance Executive	Logistics & Supply Chain	1-5
6	R6	Logistics Manager	Logistics & Supply Chain	1-5
7	R7	Corporate Finance Executive	Sales & Marketing	1-5
8	R8	Engineer	Consulting	1-5
9	R9	Corporate Finance Executive	Consulting	1-5
10	R10	Engineer	Logistics & Supply Chain	1-5
11	R11	Corporate Finance Executive	Manufacturing	1-5
12	R12	Senior Manager	Sales & Marketing	6-10
13	R13	Logistics Manager	Logistics & Supply Chain	1-5
14	R14	Sales/ Marketing Executive	Logistics &Supply Chain	1-5
15	R15	Engineer	IT Services/ Software	6-10
16	R16	Senior Manager	Logistics & Supply Chain	5-10
17	R17	Senior Manager	Logistics & Supply Chain	>10
18	R18	Logistics Manager	Logistics & Supply Chain	1-5
19	R19	Senior Manager	Logistics & Supply Chain	1-5
20	R20	Consultant	Consulting	1-5
21	R21	Engineer	Logistics & Supply Chain	1-5
22	R22	Corporate Finance Executive	Consulting	1-5
23	R23	Senior Manager	Manufacturing	6-10
24	R24	Senior Manager	Manufacturing	6-10
25	R25	Engineer	IT Services/ Software	1-5
26	R26	Senior Manager	IT Services/ Software	1-5
27	R27	Logistics Manager	Construction/ Real Estate	6-10
28	R28	Marketing Executive	Food & Beverage	5-10
29	R29	Senior Manager	Financial Services	>10
30	R30	Logistics Manager	Logistics & Supply Chain	1-5
31	R31	Logistics Manager	Logistics & Supply Chain	6-10
32	R32	Engineer	IT Services/ Software	1-5
33	R33	Engineer	Manufacturing	1-5
34	R34	Director/ Founder	Food & Beverage	>10
35	R35	Director/ Founder	Logistics & Supply Chain	>10

### Data Analysis

Data analysis was conducted using open, axial, and selective coding that further enabled us to derive themes and sub-themes through the process of iteration (Dezdar & Sulaiman, 2009; Strauss & Corbin 1997). Both the Glaserian and Straussian grounded theory methodologies are the most prevalent in extant literature (Matavire & Brown, 2013). The Glaserian approach is rooted in critical and rationalistic thinking, whereas the Straussian approach is pragmatic and can identify the phenomenon through interaction among participants. A thematic analysis of the qualitative data using the Strauss & Corbin (1997) method was adopted as it supports the development of theory based on the stories and views of participants and it provides a pre-defined coding scheme. We conducted primary, secondary, and tertiary analysis (three-layer analysis i.e., open, axial, and selective coding) since it was capable of consolidating and facilitating the raw data to meaningful themes.

Four key propositions emerged out of the data that was analysed through three stages. The first stage of the data analysis involved reading the transcripts, identifying differences and similarities between responses to the interview questions, and observing emerging themes. This stage is about conceptualisation and description of the data at basic level and is commonly known as open coding. In the second stage, we analysed the relationship among open codes leading to develop the axial codes. Second stage of axial coding of the data analysis involved further categorisation, by matching the open codes to the underlying constructs of confirmation bias. The third stage of data analysis known as selective coding employed for identifying the relationship among axial codes to map the phenomena of emerging echo chambers. Through this three-stage process of data analysis, we shed new light on the role of confirmation bias in participants involved in supply chain information processing. The coding scheme is presented in Appendix Table 8. Table 2 presents the triangulation approach, where we found the convergence through industry reports, research articles and data collected from the participants.

**Table 2:** Triangulation approach

Industry Reports	Research Articles	Data Gathered	Evolving Themes
India Today (2021) - The experiences and incidents shared by people on social media led to develop the hesitancy about Covid-19 vaccination.	Sasahara et al. (2021) - The social influence and follow personalities or topics on social media lead to the development of echo chambers later.	R9- As a consultant, I have found many executives’ referring a particular platform for their processes on the bases of success story shared by their peers on the social media, without it verified.	-Experience of others and developing own mind-set - Role of actors such as relatives and neighbours in fastening the baseless beliefs.
McKinsey & Company (2015) - The organizations are adopting the digital routes not based on their actual requirements, but their possibility of success comparing with their competitors. The organizations are adopting cloud-oriented solutions for their data that can be quickly retrieved.	Laato et al. (2020) - The misinformation spreading through social media is dangerous towards the effective response to Covid-19 response, whether it is tracing, treatment, or vaccination. Therefore, secured, and safe data needs to be verified.	R21- The social media has been witnessed in doing the role of promoting and favouring local supply chain that may occur costly later.	- Digitization -Quick and real-time information sharing -Vocal for local supply chain - Safety and security
KPMG (2021) - social media influences the consumption of data which is based on the algorithm-based mechanism to offer personalized choices based on historical search.	Nabity-Grover et al. (2020) - The social media-based consumption of data is directed based on interest of the consumer and that further deepens the confirmation bias in certain opinion of consumers.	R7- I have been posting and searching about the price dynamism in the aviation industry, and I frequently the fares of flights, and I receive the frequent email and links to flights on a particular route, that may be due to the search I have conducted recently.	-Personalized choices and consumption of data -Individual and Organizational biases
PwC (2021) - The organizations and their supply chains are influenced by different actors and their activities on different platforms apart from working in typical factory set-up.	Islam et al. (2020) - The products and services offered by organization mature as social identity over the period and that can further influence the confirmation bias leading to the development of echo chambers.	R15- We have hired a consultant for a process improvement of hydro sulphate supply chain and the communication among customers, the consultant shared his experience working with similar organization and its response on social media in terms of effectiveness of communication.	-Consulting organizations -Employees as actors -Companies own image

## Key Findings & Emergent Themes

This section presents key findings under four themes that emerged from the data, namely, environmental setting, actors, mechanism, and outcome. Each of these themes are discussed in the context of supply chains and form the basis for the proposed framework of SMIP and the emergence of echo chambers.

### Environmental Setting

The surroundings and fragmented society derive the unequal status and economic displacements in every part of the world (Bessi, 2016). The society represents individuals with democratic and republican mind set from political discourse view. Democrats and republicans develop a ground for opposite views with their own belief system and philosophy (Neiman et al., 2016). The role models, influencers and benchmarks advance the environmental setting. Additionally, the persuasion of social and human ideas, social security, government policies and economic principles lay the foundation in the interaction of diverse stakeholders on different platforms influencing the surrounding factors (Barnett, 2007). These adjoining factors further rise to the diverse culture that has a close relationship towards the active participation and supporting the diverse activities in a supply chain. Table 3 displays the related quotes and codes to environmental setting that represents the formation of echo chambers.

**Table 3:** Sample of quotes and coding for the environmental setting

Selective Code	Axial code	Open code	Quote
Environmental setting	Expectation	Social media influence the expectations from a particular supply chain	*One of major benefit of employing social platforms is to serve and solve customer queries to enhance the level of accountability of the organisations. Using Twitter like medium can expose the brands, how they respond, and it becomes a tool to ensure effective value chain (R5).*
	Experience	To enhance both online and offline experiences with customers organisations take the route of social media	*Social media has been observed as an experience platform, where satisfied and unsatisfied individuals share their views and can impact positively and negatively. Additionally, offline, and online experiences are shared in the support or against the supply chains and users of social media develop their beliefs based on others experience (R10).*
	Information platforms	Internet, blogs, and other modern platforms help customer and organisation to organise its supply chain more efficiently.	*In many firms the attention is paid to core manufacturing and distribution for their supply chain. This turns their operations disjointed and less efficient. The modern platforms such as social media can be employed to get connected and meet the diverse needs of customer through greater visibility and control (R21).*

### Actors

Social media generates large volume of data that is influenced by agents or actors. These agents (e.g., partisan, and bipartisan) work around the specific features and characteristics that focus on certain pain points ranging from individuals to large entities such as supply chain (Shin & Thorson, 2017). For instance, Facebook uses the private data of users to expose them with large amount of content (Bessi, 2016). The companies or product specific characteristics distract the users from the fundamentals through social media and it serves as a society-fracturing tool. The consulting firm’s belief as an actor strengthens when their success or failure stories are witnessed over social media (Anand et al., 2007). The belief system further gets a strong bond when other similar entities succeed or fail that lead to make their belief system more extreme (Teo et al., 2008). Supply chain interaction with social media lead information exposes it to numerous topics and global events. For example, Covid-19 directed practitioners to take certain actions to derive the business operations that define the social identity of the supply chain, which plays a critical and influential role in supply chain decisions. Table 4 displays the related quotes and codes for actors that contribute to the formation of echo chambers.

**Table 4:** Sample of quotes and coding for actors

Selective code	Axial code	Open code	Quote
Actors	Consulting firms	Similar supply chains adopt different strategies to drive business outcome influenced by consulting organisations	*There are handful of companies in supply chain and logistics those help their customers in developing the expertise, skills, and knowledge to take appropriate decisions. Due to the diverse spectrum of knowledge and understanding the firms operating in same business have opposite ways to drive the business right from the alignment of information systems, digital technologies to margins. For example, some supply chains are market driven, whereas some drives the market (R24).*
	Companies and products	Companies and products as key stakeholder can drive the supply chain in different directions	*Involving stakeholders in the design process help to answer the issues in design and materials, whereas there are other successful companies they design the products by themselves, and customer accept it as a thrill. One system favours the customer perception driven by company status, products they offer, celebrity associated, whereas other products and company market performance is driven by customer perception (R8).*
	Social identity	The supply chains establish an orientation to establish their identity based on the belief system of a group	*The organisation, strategy, design, tools, and enablers in a supply chain define the architecture and degree of robustness that can be viewed differently from the experiences ranging from consumers and providers have had. Covid-19 has exposed the key actors’ importance in defining the expression of diverse supply chains on the parameters of leanness, agility and resilience emerged from a group of advocates (R13).*

### Mechanism

In the age of social media not only in individual, but also in business to individual and business to business interactions, the personalisation is pervasive (Neves et al., 2014). The personalisation facilitates the higher user efficiency and greater revenue to the platform by providing relevant content to the user needs. Feeds, group membership, product recommendation and e-advertisement pervade the system towards personalization (Tim et al., 2018). Data and content selection algorithm consider the user’s characteristics, activities on social media and interests based on past behaviour to display the personalised list of content (Stepan et al., 2016). The personalized content and news feed along with other sources can lead to filter bubble in developing a tunnel vision for individuals to groups (Pariser, 2011). This way practitioners as well as systems like supply chain develop their ideological bubble. On the other hand, the choices that individuals make on social platform facilitate group and systemic biases to polarise the belief system (Dubois & Blank, 2018). Content is categorised into diverse groups based on sensitive attributes viz. some stories may have a particular political orientation (liberal or conservative) or a topic that can impact a particular sector of the business or supply chain.

The objective of data selection algorithm is to match the user needs to maximise the positive feedback; hence it must learn and organise about the topics that the user is most interested in. Table 5 displays the related quotes and codes for mechanism that represents the formation of echo chambers.

**Table 5:** Sample of quotes and coding for mechanism

Selective code	Axial code	Open code	Quote
Mechanism	User choices	Practitioners of supply chain are choosing the information based on their requirement	*We have been getting consulting services from a top firm due to their success with other players that the service provider firm have posted on LinkedIn. We have been searching for a firm who can help in developing and designing the digital architecture for our supply chain to address the disruptions caused by Covid-19 (R16).*
	Algorithmic personalisation (Filter bubbles)	Information displayed is based on personalised search queries and platform algorithm provides the content based on diverse parameters.	*In Covid we were tasked to identify the risk nodes for risk assessment in our supply chain. When I browsed the LinkedIn and Internet in my head office, it was showing the information and communication technologies as priority, when I searched on next day at my home, then it was showing the severity, occurrence, and detection for risk management. I have observed the differences in information display on social media when I browse through laptop or mobile or through different browsers, however it was about the SC risk management (R2).*
	Biases (Individual, group, and system)	WhatsApp platform communication by individual create a group bias and reflect in the entire supply chain system.	*In our company sales people always influence forecast in supply chain through WhatsApp to ensure the enough availability of finished goods to market, whereas operations group who are responsible to manage supplies, inventory and production favour smooth demand and avoid costly affairs involved in the process. To overcome this a new system of single business numbers have been stated that may have other active biases (R32).*

### Outcome

The digital economy and environment have steered the multitudes of information platforms (Tim et al., 2021). The practicing managers in supply chains should examine and analyse large amount of information and by doing so they may favour or develop certain channel of information in their day-to-day operations. This selection of information channels creates the risk of online echo chambers that reinforces the pre-existing biases and beliefs (Flaxman et al., 2016). The choice of social media platforms or information channels have a significant impact on consumer choice and therefore, exposes risk to stakeholders of supply chain. The familiarity and inclination with a social media source of information outplays as compared to its inherent reliability and trustworthiness (Dwivedi et al., 2018; Butler & Gray, 2006). This is observed by the fact that even though witnessing an increase in computerisation and availability of information around 33 % of the companies have not analysed their supply chains for the potential sources of disruption (Gomber et al., 2018). The remaining 67 % of the companies have developed their echo-chambers based on the success and failure stories pre and during Covid-19 to ensure the continuity of their business. Covid-19 has compromised many individual accounts on social as well as personal space that indirectly influence the supply chains and hence consider investing in strengthening cyber security, increasing flexibility, strengthening local supply chains and identify the risk nodes for better response during emergency situations (Spanaki et al., 2019). Table 6 displays the related quotes and codes to outcome that represents the formation of echo chambers.

**Table 6:** Sample of quotes and coding for outcome

Selective code	Axial code	Open code	Quote
Outcome	Digital architecture	Digitisation is beneficial for one sector can be helpful for me and we can follow the same steps as others.	*Many industries have been shared their success stories on LinkedIn of digitisation and its importance for supply chain during Covid-19. I represent the construction industry and I think a robust digital network will help our company also to reduce the time of completion of projects and efficient usage of materials along with resources (R18).*
	Real-time information	Real-time information tracking will enhance the visibility and transparency.	*Many supply chains lack in having an overall view of information sharing across their partners and hence fail to fulfil the gap of supply and demand. In today’s global and fast changing market scenario the real-time information tracking help developing competitive edge over others (R14).*
	Flexibility	Supply chains needs to be flexible to match the performance requirements in dynamic environment.	*Our company has closed one of its main product manufacturing and started manufacturing the masks and later we developed a hand sanitiser unit also during Covid-19 and partnered with third party for the distribution part, this helped us to maintain the performance of the organisation in right direction. Upon sharing our success over social media other have also tried, but very few succeeded (R28).*
	Safety and security	There can be an increase in spending of secured systems regardless of sectors to ensure data and networks are safe.	*During Covid-19 as compared to physical security, the cyber security has become more crucial due to events happening around the world. For most of the supply chains the data is on cloud and networks which is communicated regularly. Therefore, disaster recovery plan from operational and user perspective should be in place by organisations (R16).*
	Regionalisation	Focusing local supply chain can help us to ensure business continuity.	*We were procuring the raw materials before Covid-19 from multiple parts of the world due to its cost competitiveness and quality. I have read few reports on internet, and it indicates that due to increased prices, shortage of raw materials and lot more time consumption during Covid-19 it will be difficult to survive and that has led us to draw our focus from globalised souring to local and maintain the desired resilience (R33).*
	Mitigate the risk	Risk identification and developing measures to mitigate to ensure smooth flow.	*We have focused on business continuity strategies and diversified our supply chains from geographic perspective to decrease the supply chain risks based on reports published on internet. We have developed the system that diversify our system and prioritise the probability and its impact (R7).*

Following the thematic analysis, the study captured different open codes from the data of interview. After multiple iterations, back and forth, we recognized the axial codes those were further integrated to develop selective codes. In summary, our study identifies four selective codes presented in Table 7.

**Table 7:** Summary of findings

Selective Code	Axial Code	Meaning of different Open Codes
Environmental setting	Expectation	Social media influence the expectations of varied consumers based on experiences they have in physical, online, and internet-based platforms.
	Experience	
	Information platforms	
Actors	Consulting Firms	Different consulting organizations offer different solutions to the concerns of customer engagement to improving their supply chains, that further influence the positioning of companies and products in the form the celebrity endorsement and market performance. The social identity of a product/organization is perceived based on experiences they have.
	Companies and products	
	Social Identity	
Mechanism	User Choices	The executives of upstream and downstream access the social media information based on their requirement and that is further advanced based on algorithmic personalization when it comes to ultimate consumer and leads to filter bubbles. The specific groups on different platforms may create certain biases or myopic view to certain processes in the topics of their interest.
	Filter bubbles	
	Biases	
Outcome	Digital architecture	Success of a digital architecture for one business may be helpful for my business based on LinkedIn sharing. The flexible layout of the company is directly related to the capability of a company and not the copy pasting ensure the success of business activities. Many organizations are concerned about the safety and security of data along with regionalization of their supply chain activities to mitigate the risk, due to several stories shared on social media by practitioners and consulting organizations, which may not be the case.
	Real-time information	
	Flexibility	
	Safety and security	
	Regionalization	
	Mitigate the Risk	

## Propositions and Framework for SMIP in Supply Chains

In this section, we developed four propositions based on the thematic analysis of the data. The propositions demonstrate the contextual complexity of supply chain information sharing and the impact of information sharing that is highly nuanced.

- Proposition 1: The off-line and online experiences of diverse stakeholders and degree of exposure to social media set the diverse expectations from a supply chain and influence the partisan and bi-partisans orientation.

The offline experience of individuals, professionals and organisations acts as base to model their cognitive behaviour in online platforms and social media (Arnott, 2006). Users expect the content in line of their expectation and experience on internet and that further influence their beliefs. In the words of R30 (Logistics coordinator with 3 years of experience from logistics and supply chain), “*Earlier it was expected that internet and other social media can provide greater transparency by offering more content, however with varied expectations and experiences of stakeholders has developed a different kind of environment for users and their thought process”.* Many social media users consume the information without seeing the source and develop their theories for the topic of their interest or related to it (Westerwick et al., 2017; Zamani & Pouloudi, 2021). Social media is a mix of genuine and fake content posted by related individuals, groups or organisations and it is very difficult to make out and filter the true ones (Tim et al., 2018). In the words of R9 (Analyst with 3 years of experience from consulting), *“Many of the supply chain decisions are aligned with the incidents reported on social media to save the image of an organisation. Additionally, many consumers on the experience of their friends or relatives opt social media to complain or connect to company in case of product failure and this has been very frequent during Covid-19 due to face-to-face restrictions. In some cases, the influential people amplify the voice of actual complainer*”. This way the exposure and expectation from social media platforms including online and offline experiences act as a basis to develop a certain belief system and influence the activities of value chain.

- Proposition 2: Supply chain consulting firms create and develop new forms of knowledge and information that help in displaying the social identity. The products and service behaviour are tracked to recognise the consumer choices for personalised content to be offered.

In the age of information many functions of a business are using social media to understand the market trends and reaction to a specific offer (Felix et al., 2017). It has become more obvious for supply chain and related professionals, since facilitating the consumer is one of the key objectives and most of the consumers are present and use social media for many decisions that further influence the activities of a supply chain (Fu et al., 2013). In the words of R26 (Logistics manager with 10 years of experience from construction), “*The performance of a supply chain is measured, how the product and organisational efforts are recognized and perceived by real consumers, therefore to track the product performance and resolve the complaints we are connected to our consumers through social platforms such as Twitter, Facebook and LinkedIn and also we consider consulting firms advices and piece of information shared on their websites regarding changing needs of a supply chain*”. The expectations of customers are also advanced by some of the consulting firms indirectly and therefore the organisations map their social identity to match the customer expectation and the cluster they belong to (Sharif, 2002). In the words of R31 (Logistics manager with 10 years of experience from logistics and supply chain), “*The supply chains design their networks to avoid the role conflict and maintain intergroup relations due to the large players involved in delivering a product and offering aftersales services, therefore the supply chain develops a specific social identity to create the user perception”.* The network structure and architecture can act as an actor to influence the user choices on social media. These actors exchange and develop their knowledge based on their feeds.

- Proposition 3: User choices trigger the algorithmic personalisation that form the filter bubbles and vice-versa. These filter bubbles influence the individual, group, and system biases and vice-versa and it creates the echo-chambers along the supply chain.

The opinion and existing knowledge gained through websites, blogs and other forms of internet help forming the choices of the content a consumer want to view (Zhao et al., 2020). This represents the polarisation cycle from the view of confirmation bias that is pervasive. In the words of R35 (Director with more than 10 years of experience from logistics and supply chain), *“We use multiple personalisation approached on the basis of user clusters and their search history to enhance the loyalty of the user to our portal that further advanced the product recommendation through genetic adaptive architecture for profiling”.* This personalisation creates the filter bubble for individuals and groups (Garrett, 2009; Kapoor et al., 2018). In the words of R20 (Consultant with 3 years of experience from consulting), “*In one of the experiences with my client, I offered applications from different vendors for enterprise resource planning, to my surprise and save on time, they advised me to describe the product offered by Oracle. Two years later, when I visited another organisation from the region, they quoted the earlier organisation success and opted for Oracle”.* Therefore, the polarisation occurs due to the individual, group, and system biases.

- Proposition 4: The user’s choices, experiences and algorithmic personalisation influence the confirmation bias that further develop the echo chambers in supply chains. As a result, some supply chains are focusing more on digitalisation or monitoring real-time information, where some others are investing heavily on cyber security, risk mitigation and regionalisation.

Due to the exposure to large amount of data in supply chain processes, tracking and monitoring of customers and their pain points through social media becomes a complex task (Manavalan & Jayakrishna, 2019). There has been huge wave of data and that has been advanced during Covid-19 due to more time spent by public on internet and social media platforms as well as companies interacting with their customers (Nabity-Grover et al., 2020). In the words of R10 (Engineer with 3 years of experience in logistics and supply chain), “*The non- face to face interaction to ensure business continuity has turned many organisations to automation and to strengthen their digital architecture, whereas the players those could have avoided this investment on digitisation have also invested and some of them resulted into low return of investment, thus creating an echo chamber with respect to digitisation”.*

*S*upply chains have been found referring to the websites and reports of consulting companies and sharing few success stories have focused on enhancing the flexibility and approaches for identifying and mitigating the risk at different nodes (Anand et al., 2007). With the digitisation, there are more transactions online, therefore online tracking of real-time information is critical. In the words of R25 (Engineer with 5 years of experience in IT services), “*With the increase of digital payments and online tracking the security and safety of the systems is critical and have been advised by a consulting firm to spend a specific amount of the revenue in ensuring the networks are secure and we are proceeding in that direction”.* Separately from this, the focus on local supply chains have been emphasised and realised due to time, safety, and cost of sourcing from global supply chains.

**Fig. 3:**
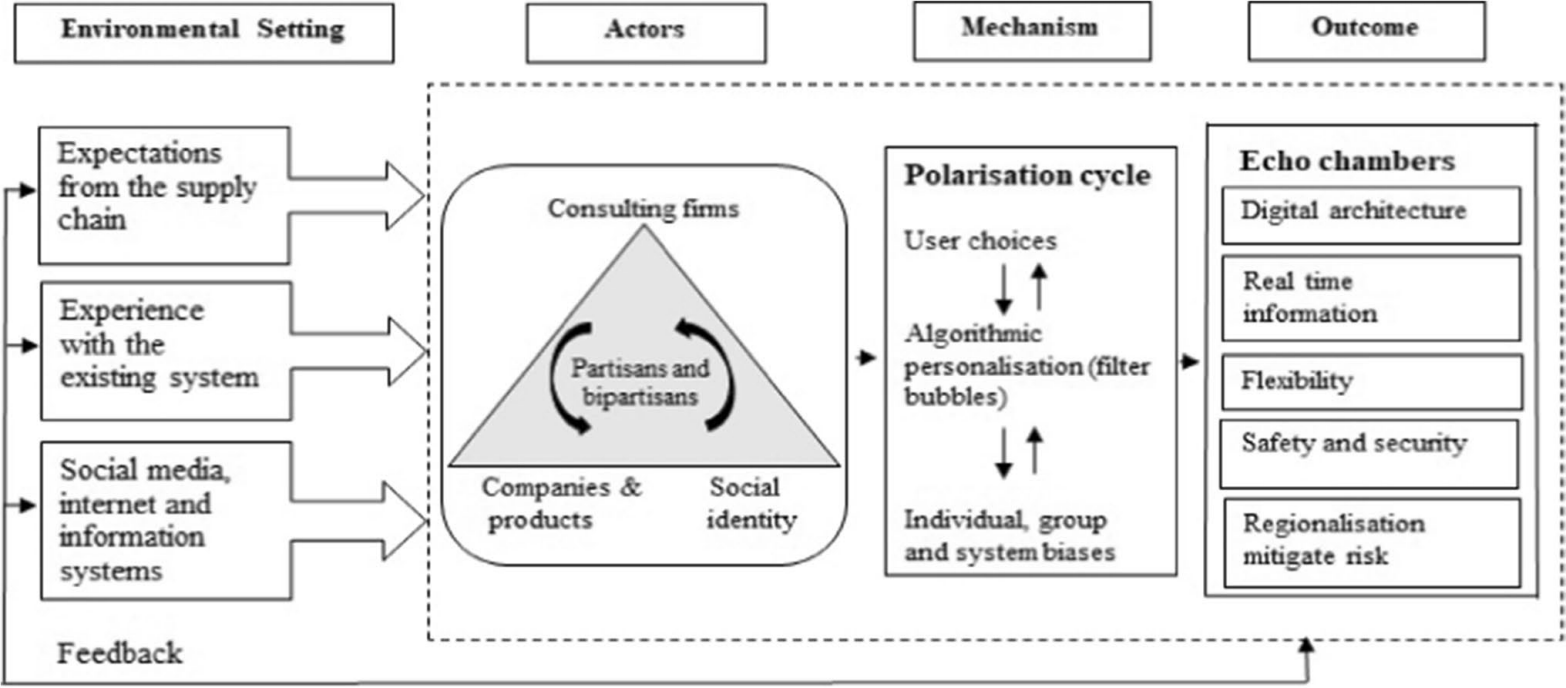
Proposed framework for social media induced polarisation in supply chains

Building on the emergent themes and propositions, a conceptual framework of SMIP and the emergence of echo chambers is presented in Fig. 3.

## Discussion

Research has recently addressed the implications of SMIP in the context of supply chains highlighting issues in decision making (Sharma et al., 2020), risk management (Fu et al., 2013; O’Leary, 2011), sentiment (Wuebben, 2016; Zadeh & Sharda, 2014). To date however, limited research exists on understanding how manifestations of confirmation bias contribute to the development of ‘echo chambers’ in the context of supply chains. A context where it has been acknowledged that participants are highly vulnerable to developing echo chambers through confirmation bias (cf. Boutyline & Willer, 2017; Orji et al., 2020).

Drawing on contemporary literature, we frame the theoretical and empirical contributions of this study (Ågerfalk, 2014; Corley & Gioia, 2011; Zamani et al., 2020). This is attained in several ways. The most salient theoretical contribution of this study is to use the confirmation bias and echo chambers as a lens to study SMIP in the context of supply chain during the Covid-19 pandemic. This is an important contribution as studies linking confirmation bias and echo chambers to supply chains remain under studied (Fu et al., 2013; O’Leary, 2011; Orji et al., 2020). This study also contributes to addressing the grand challenges of IS research (Becker et al., 2015; Winter & Butler, 2011), by specifically focusing on important socio-technical challenges such as effective IS for emergency management, and leveraging knowledge from data, with the related management of high data volumes (Becker et al., 2015). As this study builds on extant SMIP literature, it makes an empirical contribution by extending the generalisability of SMIP to provide novel insights that have not been reported in previous studies. This is achieved by identifying four themes (e.g., environmental setting, actors, mechanism, and outcome) and developing a set of propositions in the context of supply chains that may affect the extent to which confirmation bias facilitates creation of echo chamber in supply chains.

### Implications for Research

This research builds on the cumulative body of knowledge related to SMIP, by rigorously identifying four themes (i.e., environmental setting, actors, mechanism, and outcome) and developing a set of propositions that provide rich qualitative insights. These propositions, which were developed through the lens of confirmation bias, can enable researchers from a range of disciplines to assess them through hypothesis testing. The proposed framework conceptualises the complex phenomenon of SMIP, which in turn provides a structure for researchers to examine the four themes collectively or individually, and in different contexts. This study extends previous studies by demonstrating that there is a reciprocal, reinforcing relationship between confirmation bias and echo chambers that accelerates SMIP, as opposed to just the social media platform itself. Our study displays the tendency to search, recall and favour the information with reference to existing beliefs and experiences that professionals have online and offline. Further, our study contributes to SMIP literature by presenting the relationship among different aspects developed based on filter bubbles created and user choices leading to polarized in supply chains. Other studies in the literature indicate the other forms and ways of forming confirmation bias that leads to emergence of echo chambers. For instance, Sasahara et al. (2021) pointed out that minimum social media exposure also influences the thought process and developing a network of choice by un-following people of a different mind-set, leading to homogenous communities and echo chambers. Apart from developing polarization and echo chambers, confirmation bias reveals the personal traits of the individual, group, and system specifically in dynamic times such as Covid-19. Our study has unveiled the structure of elements that lead to the creation of echo chambers across the supply chain.

### Implications for Practice

The research provides further evidence to support supply chain practitioners and members of online communities to reflect and reassess their pre-existing biases (Parra et al., 2021) and how their choice of social medial platforms can positively or negatively reinforce this deep-seated belief system. Before adopting, referring, and developing an opinion about the information on social media that can directly or indirectly influence the supply chain, it is imperative to analyse and understand (i) the *source* of information (ii) *validation* of information, and (iii) *openness* while considering the information. Managers need to increase awareness about highly influential nature of online and offline information in terms of employees’ acceptance of information received via social media and their role as consumer and producer of this information further. Managers need to understand the highly complex, contextualised environment of supply chains due to the large number of actors involved in the ecosystem, perception, orientation of the organisation, type of products and what kind of social identity their supply chain is displaying to online consumers. The managers can identify the nodes of filter bubbles and clusters of echo chambers in their supply networks by employing the proposed framework to solve the issues occurring in their day-to-day operations. The practicing managers, governments and consumers of the information can work together to solve the problems of fake information and developing the perspective on that basis. The technology and social media companies can come together in developing technologies those can identify the fake information and reduce the incentives for the beneficiaries of misinformation on different platforms. The lack of appropriate and precise information can further create information vacuum that offer space for misinformation to circulate. Managers should have a strong focus on the scope of developing echo chambers especially when there is limited information about what is not known. Finally, individuals need to be informed about the implications of dispersed information and how it can lead to a ‘rose tinted’ view of the real-world.

### Limitations and Future Research

We acknowledge this study has two limitations, which also offer directions for future research. First, the study was conducted during a global pandemic that behaved like a ‘once-in-a-century pathogen’ (Gates, 2020) which led to restricted movements due to national lockdowns and heightened emotive discussions around its source and solution. Future research could consider contextual factors such as the role of organisational culture which has not been adequately explored in this context, and there is a need for research linking social media technology to national culture (Gupta et al., 2018; Gupta et al., 2019). Second, the utility of the proposed framework for SMIP in supply chain has not been evaluated. Future studies could adopt a design science approach to assess its utility in the context of supply chains or extend it to other social settings to identify the sources and consequences of echo chambers. Despite these limitations, it provides direction for future multidisciplinary research.

## Conclusions

This study draws on confirmation bias and echo chambers to advance understanding of how manifestations of confirmation bias contribute to the development of echo chambers in supply chain information sharing during the Covid-19 pandemic. The findings demonstrate that there is a reciprocal, reinforcing relationship between confirmation bias and echo chambers that accelerates SMIP, as opposed to just the social media platforms itself. Concluding, this study advances understanding of the architecture of confirmation bias that leads to echo chambers, not just in the context of global pandemics, but also in national and international events that can have devastating effects on societies.

## Appendix

**Table 8:** Open and axial codes

Coded Interview Data	Open Code	Axial Code
The behavior shown on social media platforms is captured by algorithms that further filter the content delivery to users. For instance, on a social media platform you are presented with the content generated through your close contacts such as family members and friends (R29)	Online behavior is determined based on search queries executed and algorithmic mechanism developed	Algorithmic personalization
Social media uses the algorithms those arrange the posts on the bases of relevancy instead of publishing time. Through this characteristic, social network prioritizes the feeds of user by the likelihood that they want to explore and see (R6)	Using algorithms social media offer the personalized content to users.	
The organizations promote their brands through social platforms, and when consumer endorse the related brands on the platform, they expect social platform to be used to solve their problems, since it is one message away and company will reach out to them for the concern they have (R25).	Social media raises the expectations of consumers in a value chain	Expectation
Most of the times consumers take social media route to communicate to a company and authorities when they need to reach out to someone from the organization. This sets the expectations of other consumers to have visibility and transparency in the supply chains (R4).	It is easy to communicate through social media and enhances transparency.	
Organizations and supply chains today are exposed to multiple risks; therefore, it becomes apparent for firms to include the practices such as cargo insurance, diversified supplier base, maintain transparency and ensuring supplier quality (R17).	Developing appropriate supply chain risk management practices.	Mitigating the risk nodes
The globalized supply chains carry sensitive information and increased complexity in the supply chain design creates more potential failure points and greater degree of risk. The firms can develop a risk management framework based on identifiable and non-identifiable risk for better control in their supply chain (R22).	Identify the potential failure points and degree of risk to develop a risk management framework.	
The product type has a great influence in the design of supply chain network and their impact on different stages that it follows. The organizations also influence or converge the different product’s supply chain structure and strategy resulting into similar distribution and logistics patterns (R8).	Design of products and working style of organizations influence supply chain.	Companies and products
Transportation is one of key element of a supply chain and the cost of transportation and inventory is the function of the way product is designed or improved over the period. Apart from meeting customer needs, the organizations have to consider packaging, concurrent processing and standardization in the supply chains (R27).	Parallel processing culture and efficient transportation are driven by appropriate product design.	
Social media do not discriminate between true or false statements and lack in presenting the context in which the post or experience has been shared. This inability of social media pushes many individuals to believe or not and develop their belief system on the post shared by others (R10).	Inability of social media to filter the information in right spirit viewed by many individual users.	Experience
The customers and providers both prefer social media platforms for quick communication. However, in the era of pandemic such as Covid-19, the fake news about national and regional lockdown fueled the panic buying of grocery products, which further forged their belief based on their experience of previous lockdowns and ensure their food security (R3)	Consumers and providers use their experience and beliefs on social media for taking decisions.	
The supply chain events such as disruption of fuel supplies during the months of April, May and June 2020 have been shared widely on social media platforms and such disruptions impacts the cost of it. Therefore, social media platform can develop a mindset towards a supply chain based on activities shared frequently (R13).	Information sharing pattern on social media platform about the supply chain activities develop a mindset of consumers.	Social face/identity
The way companies respond and interact with consumers develop the image of a brand and organization. This image development is a function of capturing information in different supply chain events online and keeping everyone update with current situation (R23).	The response to online customer queries helps firms to develop their social identity.	
Information flow is one of the critical components across the value chain, where multiple partners exist in both upstream and downstream. The consumers today develop their expectation about quick and real time information based on experience with other brands or organizations (R14).	Expecting real time information based on their experience with other brands.	Real time information
Companies and users prefer the social media as a platform to make distant communication reachable in very short period that is transparent and visible specifically during Covid-19. This also help organizations to save on time and cost which is very crucial for any business (R26).	Saving on time and cost through sharing information on social platforms.	
Many users use malware via emails usually but can also be spread through social media platforms. Many users get looted through their Facebook or Instagram accounts through the exposure to hidden links and lucrative offers online (R15).	The hidden and fishing links lead to compromising online security and safety of Individuals.	Security and Safety
Since organizations are taking the fastest and cost-effective route for communicating with customers, it also poses the threats such as cyber security due to huge data shared on social media related to different events. Therefore, organizations need to have better control on weak points of social media usage (R9).	Online data sharing poses the risk of cyber-attack leading to collapse a system.	
We have considered the services for designing our supply chain during Covid-19 based on the experience shared by our competitor on LinkedIn. We were quite satisfied with the elements that we were looking for were within the capability of the consulting firm that we have shortlisted (R16).	Companies choose the information based on their requirement	User choices
The actual users and marketers will differ in their choices while selecting the content on social media. The unconscious errors in the mind guide the view, perception, interpretation and making certain decisions by choosing the information on social media platform (R29).	The users will use unconscious belief system to view, perceive and select the information on social media	
Apart from connecting with multiple stakeholder’s social media helps in digitizing the supply chain that helps beyond developing the relationships. Digitization of processes and linking it to social media help organization to share risks and allocate vulnerability quotient (R18).	Digitization linked to social media can help in sharing the risks in a supply chain.	Digital system/architecture
The business and supply chain operations consists of large number of players that make it possible to provide the product to customers. To ensure end- to- end transparency, every member should have access to all type of data specifically during Covid-19 pandemic. Digitizing the processes make it happen and offer real time insights (R34).	Digitization of processes offer better control and visibility.	
Blogs are an important medium to provide information to diverse stakeholder about the current happenings and promote new brands. Even some organizations link blog content on social media channels to promote their content effectively (R21).	Blogs and social media linkage can promote the content effectively.	Information and Social media platforms
The providers and users utilize social media to drive core activities of a business such sharing the information, posting the relevant updates, comments made by individuals on the content and tagging related individuals that leads to influence or develop cognitive biases in most cases (R1).	Utilization of social media platforms for processing the information	
The organizations develop their knowledge either on experience or information shared by limited consulting firms. The companies operating in the same domain may choose different set of tools and approaches to solve their problem suggested by consultants and experts working with employees of a company (R21)	Competitor firm may choose different solutions suggested by experts for the same problem.	Consulting organizations
Consulting organizations play a key role in solving the problems of industry, those are either old, complex or emerging due to environmental uncertainty. Consulting solutions are majorly based on experience and deep research and a particular problem can be solved in multiple ways and a user can select an approach based on their belief system (R7).	Consulting organizations can offer multiple solutions to a problem.	
Before March 2020 it was almost impossible to see our employees not coming to office and working from home. As Covid-19 lockdown announced, we prepared and trained our staff with utilizing Zoom and Webex platform for meetings. After few months we realized that even using these online platforms we were able to manage the business (R28).	Organizations can achieve the target performance levels meeting online during Covid-19.	Flexible approach
During lockdown the demand for other than essential items was lowered significantly and that forced many companies to opt for converting their factories to produce PPE Kits, Masks, Gloves and Hand Sanitizers, that has helped bidirectional, first to the nation in the fight against Covid-19 and second to achieve their performance target (R19)	Planning of alternate products to tradeoff between opportunity and performance target.	
Today organizations are trying hard to catch the real time information and for that reason WhatsApp and Telegram are one of the convenient ways among certain groups in the organizations. These groups behavior and the thinking style of individuals is influenced by the conversation they have over the period (R32).	Social media and quick messaging platforms give rise to create individual and group biases	Individual and Group Biases
The business activities and supply chain operations are driven by employees, and they are the function of personal, social and cognitive biases. Sometime small group of individuals form a different behavior and influence the business activities. For instance, even in a company the views of marketing and purchasing department differs according to their orientation and select biases (R35).	Personal, social and cognitive biases influence the business activities.	
Due to the shortage of raw materials amid Covid-19 crisis, many firms have strengthened their regional suppliers that can reduce the dependability on the foreign suppliers. Based on online reports, one can focus on the elements of strengthening the resilience in the local supply chain (R33).	Efficient regional supply chains can help the businesses to withstand Covid-19 disruption.	Regionalization/Local supply chains
Covid-19 has disrupted the supply chains worldwide and more has been written also on different platforms about the rise of freight prices and delay in shipments. We have witnessed the sharp rise in fuel prices when lockdown happened in the country and same is going to happen with other commodities those otherwise were imported from other countries (R11).	Regional supply chains can help in addressing the delay and cost part of supply chain during Covid-19.	
